# Inhibition of Akt activity induces the mesenchymal-to-epithelial reverting transition with restoring E-cadherin expression in KB and KOSCC-25B oral squamous cell carcinoma cells

**DOI:** 10.1186/1756-9966-28-28

**Published:** 2009-02-26

**Authors:** Kyoung-Ok Hong, Ji-Hong Kim, Ji-Soo Hong, Hye-Jung Yoon, Jae-Il Lee, Sam-Pyo Hong, Seong-Doo Hong

**Affiliations:** 1 Department of Oral Pathology, School of Dentistry and Dental Research Institute, Seoul National University, Seoul, Korea

## Abstract

**Background:**

The Akt/PKB family of kinases is frequently activated in human cancers, including oral squamous cell carcinoma (OSCC). Akt-induced epithelial-to-mesenchymal transition (EMT) involves downregulation of E-cadherin, which appears to result from upregulation of the transcription repressor Snail. Recently, it was proposed that carcinoma cells, especially in metastatic sites, could acquire the mesenchymal-to-epithelial reverting transition (MErT) in order to adapt the microenvironments and re-expression of E-cadherin be a critical indicator of MErT. However, the precise mechanism and biologic or clinical importance of the MErT in cancers have been little known. This study aimed to investigate whether Akt inhibition would restore the expression of E-cadherin and β-catenin, reduce that of Vimentin, and induce the MErT in OSCC cells with low or negative expression of E-cadherin. We also investigate whether inhibition of Akt activity would affect the E-cadherin repressors and signaling molecules like NF-κB, ERK, and p38.

**Methods:**

We screened several OSCC cell lines in order to select suitable cell line models for inducing MErT, using immunoblotting and methylation specific-PCR. We examined whether Akt inhibitor phosphatidylinositol ether lipid analogues (PIA) treatment would restore the expression of E-cadherin and β-catenin, reduce that of Vimentin, and induce the MErT in KB and KOSCC-25B cells using RT-PCR, immunoblotting, immunofluorescence analysis, and *in vitro *migration assay. We also investigated whether inhibition of Akt activity would affect the E-cadherin repressors, including Snail, Twist, and SIP-1/ZEB-2 and signaling molecules like NF-κB, ERK, JNK, and p38 using RT-PCR, immunoblotting, and immunofluorescence analysis.

**Results:**

Of the 7 OSCC cell lines, KB and KOSCC-25B showed constitutively activated phosphorylated Akt and low or negative expression of E-cadherin. Inhibition of Akt activity by PIA decreased NF-κB signaling, but did not affect phosphorylation of ERK, JNK, and p38 in KB and KOSCC-25B cells. Akt inhibition led to downregulation of Snail and Twist expression. In contrast, inhibition of Akt activity by PIA did not induce any changes in SIP-1/ZEB-2 expression. PIA treatment induced the expression of E-cadherin and β-catenin, reduce that of Vimentin, restored their epithelial morphology of a polygonal shape, and reduced tumor cell migration in KB and KOSCC-25B cells, which was the corresponding feature of MErT.

**Conclusion:**

All of these findings suggest that Akt inhibition could induce the MErT through decreased NF-κB signaling and downregulation of Snail and Twist in OSCC cells. A strategy involving Akt inhibition might be a useful therapeutic tool in controlling cancer dissemination and metastasis in oral cancer patients.

## Background

Oral squamous cell carcinoma (OSCC) is the most common neoplasm of the head and neck. Carcinoma cells accumulate a series of genetic and/or epigenetic changes and altered phenotypes during tumor progression. Loss of epithelial morphology and acquisition of mesenchymal characteristics, termed the epithelial-to-mesenchymal transition (EMT), are typical for carcinoma cells during tumor progression and correlate with the local invasiveness and metastatic potential of the tumor [1,2]. Among the mechanisms largely associated with the metastatic conversion of epithelial cells and the EMT, the loss of E-cadherin-mediated cell adhesion is prominent [3,4].

The Akt/PKB family of kinases is a downstream effector of phosphatidylinositol 3-kinase (PI3K) and is frequently activated in human cancers, including OSCC [5-8]. Recently, activation of the PI3K/Akt axis is emerging as a central feature of EMT. Akt-induced EMT involves downregulation of E-cadherin, which appears to result from upregulation of the transcription repressor Snail. Akt activity is induced by ligand stimulation of growth factor receptors such as the insulin-like growth factor-I receptor (IGF-IR) and the EGF family of receptors [9]. Ligand stimulation activates PI3K, the upstream activator of Akt, by direct binding to either the activated phosphorylated receptor or to adaptor proteins phosphorylated by receptor kinase activity [10]. Phosphoinositides generated by PI3K activity trigger activation of Akt kinases through direct binding to the pleckstrin homology (PH) domain and the subsequent phosphorylation of Akt at two conserved residues [11]. Therefore, we used an Akt inhibitor, structurally modified phosphatidylinositol ether lipid analogues (PIA) [12], that specifically binds to the PH domain of Akt.

Recently, it was proposed that carcinoma cells, especially in metastatic sites, could acquire the mesenchymal-to-epithelial reverting transition (MErT) in order to adapt the microenvironments and re-expression of E-cadherin be a critical indicator of MErT [13,14]. Therefore, it seems to be important to investigate which molecules or inhibitors could induce MErT in cancers. However, the precise mechanism and biologic or clinical importance of the MErT in cancers have been little known in *in vitro *and *in vivo *study.

The purpose of our study was to investigate whether Akt inhibition by PIA treatment would restore the expression of E-cadherin and β-catenin, reduce that of Vimentin, and induce the MErT in OSCC cells with low or negative expression of E-cadherin. We also investigated whether inhibition of Akt activity would affect the E-cadherin repressors, including Snail, Twist, and SIP-1/ZEB-2 and signaling molecules like NF-κB, ERK, JNK, and p38.

## Materials and methods

### Cell culture and reagents

KB, SCC-15, SCC-25 (American Type Culture Collection, Manassas, VA), HSC-3, HSC-4, Ca9-22 (from Dr. T. Takata, Hiroshima Univ.), and KOSCC-25B (from Dr. BM Min, Seoul National Univ.) [15,16] human OSCC cells were cultured in DMEM supplemented with 10% fetal bovine serum (FBS) and antibiotics (100 U/ml penicillin and 100 μg/ml streptomycin). Akt inhibitor PIA (SH-5) was purchased from Calbiochem (Gibbstown, NJ). Antibodies against Akt1/2, phosphorylated ERK (Tyr 204), phosphorylated JNK (Thr183/Tyr185), phosphorylated p65, p50, p38, Snail, SIP-1/ZEB-2, Twist, β-catenin, and E-cadherin were purchased from Santa Cruz Biotechnology (Santa Cruz, CA). Phosphorylated Akt (Ser 473) was obtained from Cell Signaling Technology (Danvers, MA). Vimentin was obtained from BD Biosciences (Franklin Lakes, NJ). α-Tubulin and phalloidin-TRITC were purchased from Sigma (St. Louis, MO).

### Pharmacological Treatments

OSCC cells were plated at 2–2.5 × 10^5 ^cells/well in 6- or 12-well plates in DMEM containing 10% FBS and incubated for 24 h. The medium was then changed to DMEM with 0.1% FBS, and the cells were incubated overnight. After overnight incubation, cells were treated with PIA dissolved in DMSO (5 μM) for 12 h (*in vitro *migration assay) or 24 h (other experiments). In all experiments, DMSO added to control samples had no effect on Akt activity.

### RT-PCR

mRNA was purified from the cells using the Trizol reagent (Invitrogen, Carlsbad, CA) according to the manufacturer's recommended protocol. Two μg RNA was added to RT-PCR reactions containing primers at a concentration of 0.5 μM. After a 42°C/60-min reverse transcription step, 30 cycles of PCR amplification were performed at 94°C for 30 sec, 58°C for 50 sec, and 72°C for 50 sec. PCR products were run on 1.5% agarose gels for identification. Primers used were 5'-TCC CAT CAG CTG CCCAGA AA-3' and 5'-TGA CTC CTG TGT TCC TGT TA-3' for E-cadherin, 5'-AAG CAG GAG TCC ACT GAG TA-3' and 5'-GTA TCA ACC AGA GGG AGT GA-3' for Vimentin, 5'-GGG CAG GTA TGG AGA GGA AGA-3' and 5'-TTC TTC TGC GCT ACT GCT GCG-3' for Snail, 5'-TTC CTG GGC TAC GAC CAT AC-3' and 5'-GCC TTG AGT GCT CGA TAA-3' for Sip1, 5'-GGA GTC CGC AGT CTT ACG AG-3' and 5'-TCT GGA GGA CCT GGT AGA GG-3' for Twist, 5'-GCT GAT TTG ATG GAG TTG GA-3' and 5'-GCT ACT TGT TCT TGA GTG AA-3' for β-catenin, and 5'-GAA GGT GAA GGT CGG AGT C-3' and 5'-CAA AGT TGT CAT GGA TGA CC-3' for GAPDH.

### Analysis of the E-cadherin promoter by Methylation specific-PCR (MS-PCR)

Methylation status of the CpG sites in the E-cadherin promoter region was analyzed based on the principle that bisulfite modification of the genomic DNA would convert unmethylated cytosine residues to uracil, whereas methylated cytosine is resistant to the treatment. Bisulfite modification and MS-PCR were carried out as described [17,18]. Modified DNA was amplified using primers specific for the methylated sequence (5'-TTA GGT TAG AGG GTT ATC GCG T-3' and 5'-TAA CTA AAA ATT CAC CTA CCG AC-3' and for the unmethylated sequence (5'-TAA TTT TAG GTT AGA GGG TTA TTG T-3' and 5'-CAC AAC CAA TCA ACA ACA CA-3'). 35 cycles of PCR amplification were performed at 94°C for 30 sec, 56°C for 30 sec, and 72°C for 30 sec. PCR products were run on 2% agarose gels for identification. MDA-MB-231 and MCF-7 (American Type Culture Collection) breast cancer cells were utilized as positive controls for methylated and unmethylated E-cadherin gene, respectively [19].

### Immunoblotting

Briefly, 70–80% confluent cells were homogenized with 1 ml of lysis buffer (10 mM HEPES, pH 7.9, 1.5 mM MgCl_2_, 10 mM KCl, 0.5 mM DTT, 0.2 mM PMSF) and incubated on ice. To the homogenates was added 125 μl of 10% NP-40 solution, and the mixture was then centrifuged for 30 sec at 12,000 × g. Supernatant protein concentration was determined by the Bradford protein assay (Bio-Rad, Hercules, CA, USA) using bovine serum albumin (Sigma) as a standard. Immunoblot analysis was performed as described elsewhere [20].

### Immunofluorescence analysis and confocal microscopy

Cells grown on coverslips were fixed in 4% PFA, permeabilized in 0.3% Triton X-100, and blocked for 40 min in 1% BSA/10% fetal bovine serum. The cell samples were incubated with primary antibodies at 4°C overnight, washed with PBS containing 0.1% BSA, and then reacted with FITC- or Cy3-conjugated secondary antibodies (Jackson ImmunoResearch Laboratories, West Grove, PA, USA) at room temperature for 40 min. After washing, the samples were rinsed with PBS containing 0.1% BSA, stained with 5 mg/ml 4,6-diamidino-2-phenylindole (DAPI; Sigma), and mounted. Confocal analyses were performed using an Olympus (Center Valley, PA) FC-300 Confocal Laser Scanning Microscope equipped with FITC- and Cy3- channel filter systems. All images were converted to TIFF format and arranged using Photoshop 7.0 (Adobe, Seattle, WA).

### *In vitro *migration assay

The *in vitro *migration assay was performed as described previously [21]. 5 × 10^4 ^cells were placed in the upper compartment (8 μm pore size) of the cell culture insert with or without 5 μM PIA. Medium, supplemented with 100 ng/ml IGF-I (R&D Systems, Minneapolis, MN), was added to the lower compartment. After 12 h of incubation, the cells on the upper surface of the filter were wiped out with a cotton swab, and the filter was removed from the chamber and stained with Diff-Quick stain set (Fisher, Pittsburgh, PA). The migration of the cells was determined by counting the number of cells that migrated through the pores to the lower side of the filter under a microscope at 100 × magnification. We performed the assay three times, and three randomly selected fields were counted for each assay. We used Student's *t *test to determine the significance at a level of *P *< 0.05.

## Results

### Screening of oral squamous cell carcinoma cell lines

**Figure 1:**
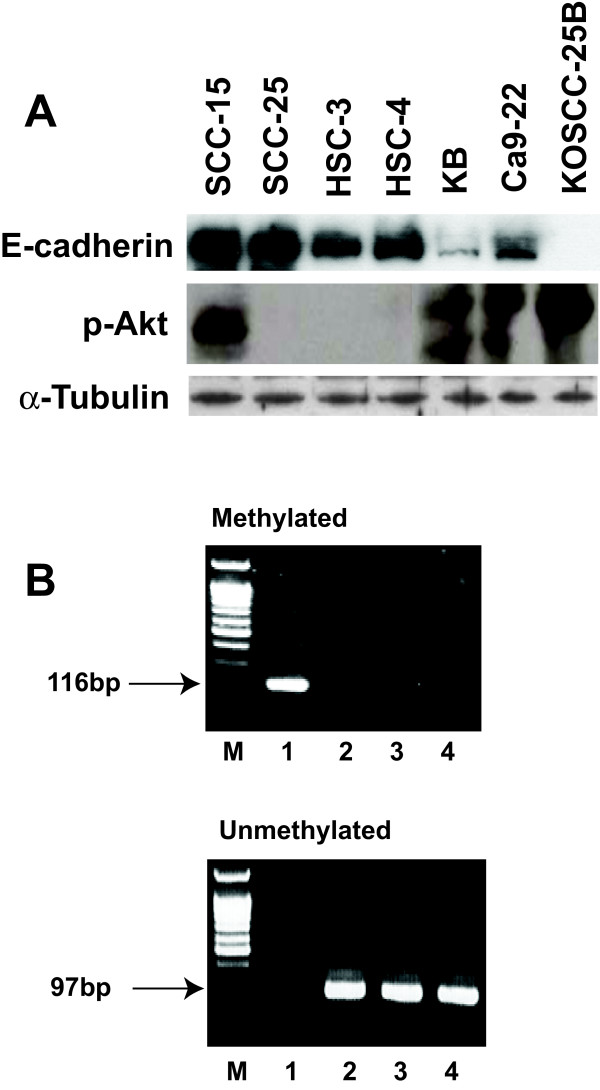
**Screening of OSCC cell lines in order to obtain a suitable cell line model for inducing MErT**. (A) Of the 7 OSCC cell lines, KB, KOSCC-25B, Ca9-22, and SCC-15 showed constitutively activated phosphorylated Akt (p-Akt). Of these four lines, only KB and KOSCC-25B showed low or negative expression of E-cadherin. (B) Methylation specific-PCR: PCR products were detected in both KB and KOSCC-25B with unmethylation-specific primer pairs, not methylation-specific ones. M, DNA ladder; lane 1, MDA-MB-231; lane 2, MCF-7; lane 3, KB; lane 4, KOSCC-25B.

We screened several OSCC cell lines in order to select suitable cell line models with the characteristics of the EMT (low or negative expression of E-cadherin) and a constitutively activated state of Akt. Of the 7 OSCC cell lines, KB, KOSCC-25B, Ca9-22, and SCC-15 showed constitutively activated phosphorylated Akt (p-Akt). Of these four lines, only KB and KOSCC-25B showed low or negative expression of E-cadherin (Fig. 1A). Because the E-cadherin downregulation could be caused by the methylation of its promoter, we investigated the methylation status of E-cadherin gene promoter in the KB and KOSCC-25B cells with MS-PCR. PCR products were detected in both KB and KOSCC-25B with unmethylation-specific primer pairs, not methylation-specific ones (Fig. 1B). These results indicate that the KB and KOSCC-25B have unmethylated E-cadherin gene. So, the KB and KOSCC-25B cell lines were chosen as suitable models for the present study.

### Effects on Akt and Akt-related signaling molecules by PIA treatment

**Figure 2:**
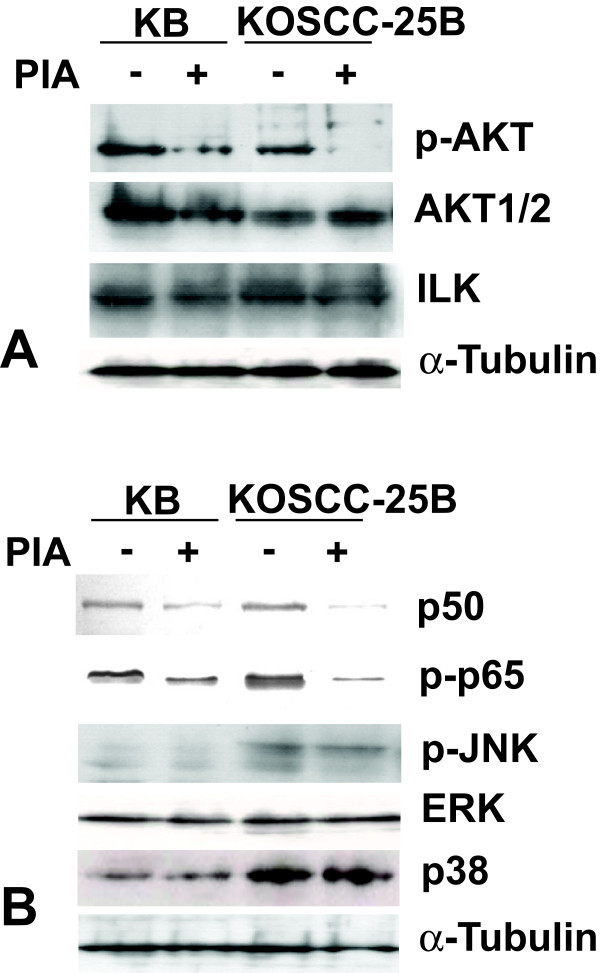
**Effects of PIA treatment on Akt and Akt-related signaling molecules**. (A) P-Akt level in KB and KOSCC-25B cells was significantly lower after 5 μM PIA treatment for 24 hours. However, Akt1/2 and ILK (upstream molecules of Akt) did not show any change after PIA treatment. (B) Inhibition of Akt activity by PIA induced downregulation of p50 and p-p65 in KB and KOSCC-25B cells, but it did not affect phosphorylation of JNK, p38, and ERK.

As expected, there were no changes in Akt1 and Akt2 protein levels in KB and KOSCC-25B cells and p-Akt level was significantly lower after 5 μM PIA treatment for 24 hours (Fig. 2A). However, ILK, upstream molecules of Akt, did not show any change after PIA treatment, indicating that PIA is a specific blocker of Akt signaling. Next, we investigated whether PIA treatment could affect signaling molecules such as ERK, p38, p50, and p65. Inhibition of Akt activity by PIA induced downregulation of p-p65 and p-50, but did not affect phosphorylation of ERK, JNK, and p38 in KB and KOSCC-25B cells (Fig. 2B).

### Effects of Akt inhibition on Snail, SIP-1/ZEB-2, and Twist expression

**Figure 3:**
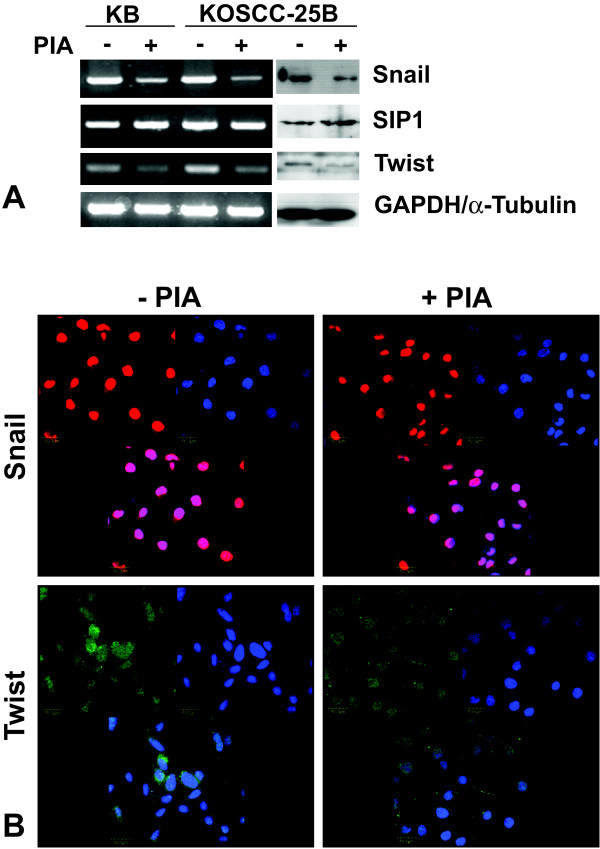
**Effects of Akt inhibition on Snail1, SIP-1/ZEB-2, and Twist expression and localization**. (A) Downregulation of Snail and Twist was detected in KB and KOSCC-25B cells by immunoblot and RT-PCR analysis. In contrast, inhibition of Akt activity by PIA did not induce any changes in SIP-1/ZEB-2 mRNA and protein expression. (B) A shift from the nucleus to the cytoplasm of Snail and Twist in KOSCC-25B cells was detected by immunofluorescence analysis.

We examined the effects of Akt inhibition on the expression of EMT-related transcription factors Snail, SIP-1/ZEB-2, and Twist in KB and KOSCC-25B cells. Downregulation of Snail and Twist was detected by immunoblot and RT-PCR analysis (Fig. 3A). In addition, a shift from the nucleus to the cytoplasm of Snail and Twist was detected in the immunofluorescence analysis (Fig. 3B). In contrast, inhibition of Akt activity by PIA did not induce any changes in SIP-1/ZEB-2 expression.

### Effects of Akt inhibition on epithelial and mesenchymal markers

**Figure 4:**
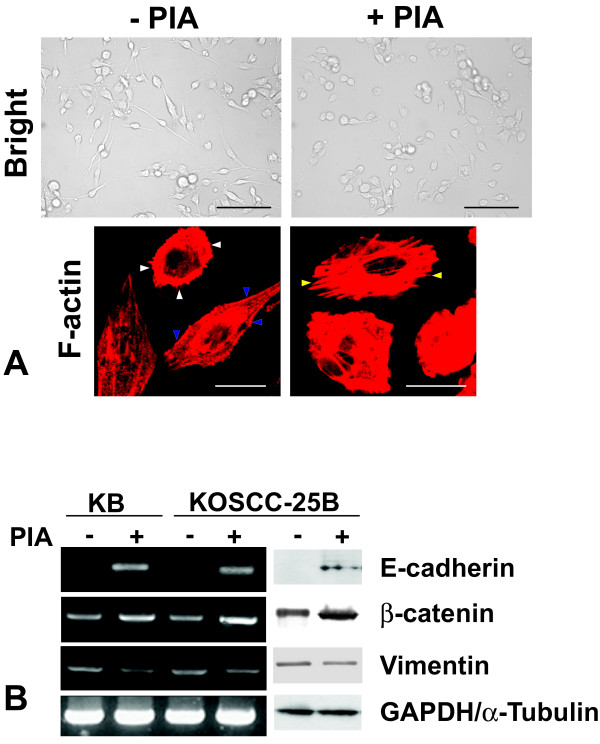
**Effects of Akt inhibition on cell morphology and the expression of the epithelial and mesenchymal markers**. (A) KOSCC-25B cells had an elongated shape, assuming a fibroblast-like appearance. In contrast, PIA-treated KOSCC-25B cells seemed to restore their epithelial morphology of a polygonal shape. In phalloidin staining, KOSCC-25B cells demonstrated circumferential, cortical actin (blue arrowheads), and actin in elongated filopodia (white arrowheads); however, no actin stress fibers were detected. In contrast, PIA-treated cells revealed an abudance of actin stress fibers (yellow arrowheads). Scale bar: 100 μm (black), 20 μm (white). (B) Inhibition of Akt activity increased the expression of E-cadherin and β-catenin, and reduced the Vimentin expression in KB and KOSCC-25B cells.

**Figure 5:**
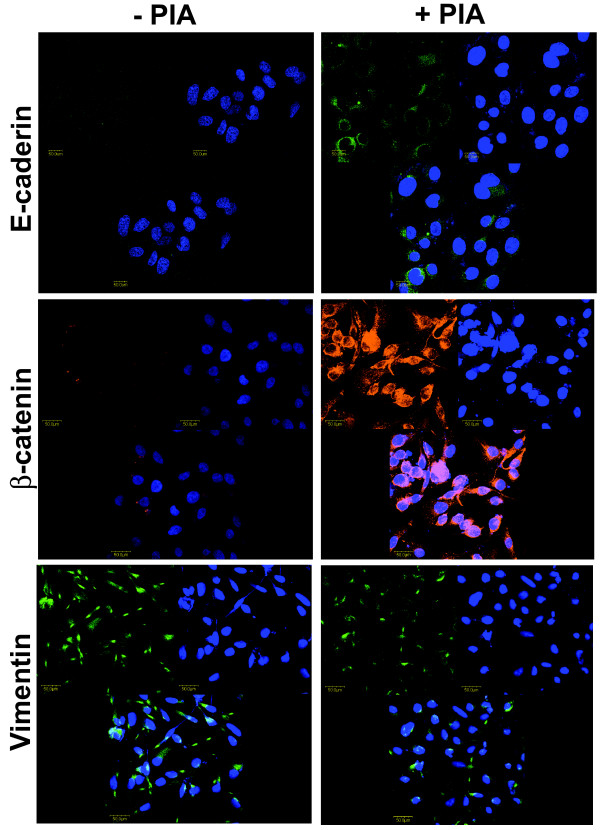
**Effects of Akt inhibition on the localization of the epithelial and mesenchymal markers**. The inhibition of Akt activity induced the localization of E-cadherin and β-catenin, and decreased that of vimentin, as seen in the immunofluorescence analysis.

KOSCC-25B cells had an elongated shape, assuming a fibroblast-like appearance. In contrast, PIA treatment of the cells seemed to restore their epithelial morphology of a polygonal shape (Fig. 4A upper panel). In phalloidin staining, KOSCC-25B cells demonstrated circumferential, cortical actin, and actin in elongated filopodia; however, no actin stress fibers were detected. In contrast, PIA-treated cells revealed an abudance of actin stress fibers (Fig. 4A lower panel). These results showed that PIA treatment of the cells induced actin cytoskeleton reorganization, which contributed to loss of the migratory phenotype. We examined whether PIA treatment could affect the expression and localization of E-cadherin and β-catenin, epithelial markers, and Vimentin, a mesenchymal marker. In accordance with the observed morphologic change, inhibition of Akt activity induced the expression in immunoblotting and RT-PCR (Fig. 4B) and localization of E-cadherin and β-catenin as seen in the immunofluorescence analysis (Fig. 5 upper and middle panel). Also, PIA treatment decreased the vimentin expression (Fig. 4B) or localization (Fig. 5 lower panel), although the change was not as prominent as that in the epithelial markers.

### Reduced migratory ability after Akt inhibition

**Figure 6:**
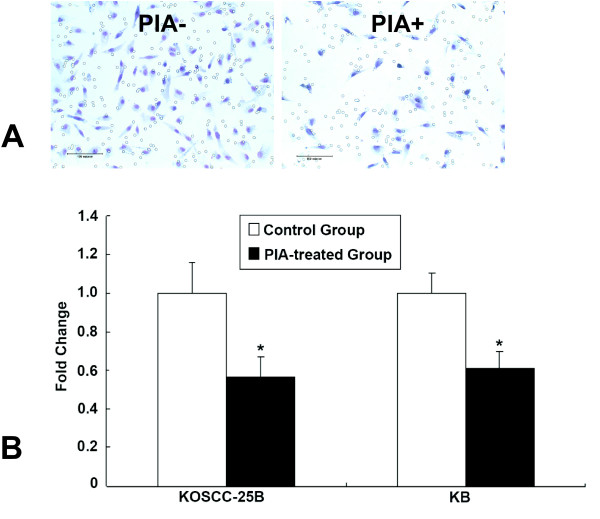
**Reduced migratory ability due to Akt inhibition**. Photomicrography of control (A) and PIA-treated (B) KOSCC-25B groups in the *in vitro *migration assay. (C) The numbers of KB and KOSCC-25B cells from the PIA-treated group that migrated through the filter were only 61.1% and 56.4% of that in control cells (*P *< 0.05), respectively.

In order to examine whether inhibition of Akt activity could affect cell motility, we performed an *in vitro *migration assay. The numbers of KB and KOSCC-25B cells from the PIA-treated group that migrated through the filter were only 61.1% and 56.4% of that in control cells (*P *< 0.05; Fig. 6), respectively.

## Discussion

During EMT, epithelial cells acquire fibroblast-like properties and exhibit reduced cell-cell adhesion and increased motility. The plasticity afforded by the EMT is central to the complex remodeling of embryo and organ architecture during gastrulation and organogenesis. In pathological processes such as oncogenesis, the EMT may endow cancer cells with enhanced motility and invasiveness. Indeed, oncogenic transformation may be associated with signaling pathways promoting the EMT [22]. Akt activation is frequent in human epithelial cancer. In our previous study [23], Akt activation in OSCC was linked to aggressive clinical behavior and the loss of histological features of epithelial differentiation. These findings are consistent with Akt directly affecting epithelial cell morphology, cell motility, and invasiveness.

Grille *et al*. [24] demonstrated that OSCC cells engineered to express constitutively active Akt underwent EMT, characterized by downregulation of the epithelial markers desmoplakin, E-cadherin, and beta-catenin, and upregulation of the mesenchymal marker vimentin. The cells also lost their epithelial cell morphology and acquired fibroblast-like properties. In addition, the cells expressing constitutively active Akt exhibited reduced cell-cell adhesion, increased motility on fibronectin-coated surfaces, and increased invasiveness in animals.

Because OSCC cells engineered to express constitutively active Akt have been known to undergo EMT, we tried to examine whether inhibition of Akt activity could restore epithelial characteristics and deplete mesenchymal features. In the present study, PIA treatment induced the expression and cytoplasmic localization of the epithelial markers (E-cadherin and β-catenin). In addition, it decreased the vimentin expression and localization, although the change was not as prominent as that in the epithelial markers. Also, the inhibition of Akt activity restored the polygonal epithelial morphology and reduced the migratory ability. This indicates that the inhibition of Akt activity could induce the MErT in OSCC cells, and that the gain of epithelial characteristic might earlier or more prominent event in the MErT of the OSCC than the loss of mesenchymal one.

Several EMT-inducing developmental regulators repress E-cadherin transcription via interaction with specific E-boxes of the proximal E-cadherin promoter [25,26]. The Snail-related zinc-finger transcription factors (Snail and Slug), the (more distantly related) repressor SIP-1/ZEB-2, and the related Snail family member δ EF-1/ZEB1 are the most prominent [27-30]. The Snail protein is one of the key molecules in the EMT and its expression is inversely correlated with E-cadherin expression in a number of cancers, including OSCC [31-33]. Accordingly, inhibition of Akt activity induced downregulation of EMT-related transcription factor Snail. However, inhibition of Akt activity did not affect the expression level of the SIP-1/ZEB-2. These data suggest that Akt signaling could induce the EMT through activation of Snail, but not SIP-1/ZEB-2, in OSCC cells.

The basic helix-loop-helix transcription factor Twist, a protein known to be essential for initiating mesoderm development during gastrulation, was recently added to the growing list of developmental genes with a key role in E-cadherin repression and EMT induction [34]. Yang *et al*. [29] demonstrated that knockdown of Twist expression by RNAi in a metastatic mammary tumor cell line prevented lung metastasis, and the high levels of Twist expression seen in 70% of invasive lobular breast carcinomas, which display many features of EMT, were inversely correlated with E-cadherin expression. However, there have been no reports on the relationship of Twist with the EMT in oral cancer cells. In the present study, inhibition of Akt activity induced downregulation of EMT-related Twist in OSCC cells. To our knowledge, this study is the first description of the participation of Twist in the EMT/MErT process in oral cancer.

**Figure 7:**
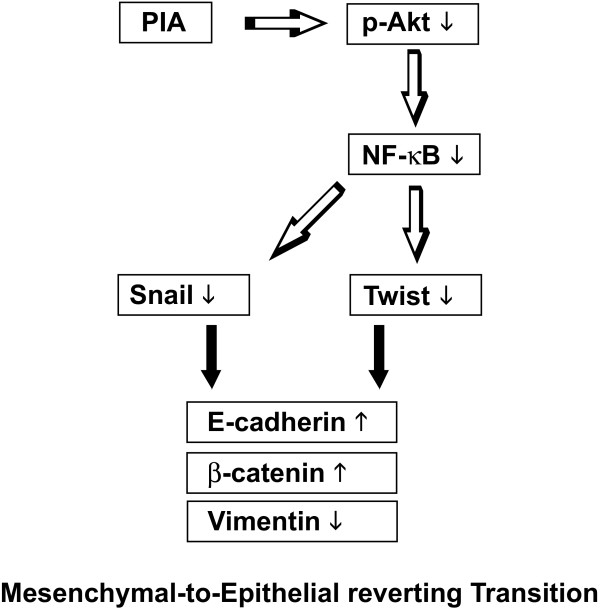
**A schematic representation of the proposed signaling mechanism that promotes MErT through the inhibition of Akt activity in oral cancer cells**.

Akt signaling has been deeply studied because Akt plays critical roles in regulating growth, proliferation, survival, metabolism, and other cellular activities [21,35]. Chua *et al*. [36] showed that NF-κB suppresses the expression of epithelial specific genes E-cadherin and desmoplakin and induces the expression of the mesenchymal specific gene vimentin in breast carcinoma cells. Similarly, Julian *et al*. [37] reported that activation of NF-κB by Akt upregulates Snail expression and induces EMT in OSCC cells, and expression of the NF-κB subunit p65 is sufficient for EMT induction. We investigated whether it could be possible in the reverse direction, which have been little known. In the present study, inhibition of Akt activity induced the MErT through interaction with NF-κB. Downregulation of NF-κB contributed to MErT. Huber *et al*. [38] showed that inhibition of NF-κB signaling prevents EMT in Ras-transformed epithelial cells, while activation of this pathway promotes the transition to a mesenchymal phenotype. Fig. 7 shows a schematic representation of the proposed signaling mechanism that promotes MErT through the inhibition of Akt activity in KB and KOSCC-25B cells. Additional study using NF-κB inhibitors could be needed in order to verify this proposed pathway.

In summary, we demonstrated that Akt inhibition by PIA treatment induced downregulation of Snail and Twist expression, upregulation of E-cadherin and β-catenin, downregulation of vimentin, and reduced cell migration, which led to the MErT in oral cancer cells. The MErT in oral cancer cells seems to be acquired through decreased NF-κB signaling. All of these findings suggest that Akt inhibition can induce the MErT through decreased NF-κB signaling and downregulation of Snail and Twist in OSCC cells. A strategy involving Akt inhibition might be a useful therapeutic tool in controlling cancer dissemination and metastasis in oral cancer patients.

## Conclusion

All of these findings suggest that Akt inhibition could induce the MErT through decreased NF-κB signaling and downregulation of Snail and Twist in OSCC cells. A strategy involving Akt inhibition might be a useful therapeutic tool in controlling cancer dissemination and metastasis in oral cancer patients.

## Competing interests

The authors declare that they have no competing interests.

## Authors' contributions

KH carried out experiments on the Akt signaling and drafted the manuscript. JK participated in the screening cell lines and migration assay. JH participated in confocal analysis and Western Blot analysis. HY participated in RT-PCR analysis. JL and SPH participated in the study design and revised the manuscript critically for important intellectual content. SDH conceived of the study, participated in its design and cooperation. All authors read and approved the final manuscript.
